# Human bone marrow mesenchymal stem cell-derived exosomes stimulate cutaneous wound healing mediates through TGF-β/Smad signaling pathway

**DOI:** 10.1186/s13287-020-01723-6

**Published:** 2020-05-24

**Authors:** Tiechao Jiang, Zhongyu Wang, Ji Sun

**Affiliations:** 1 Department of Cardiology, The Third Hospital of Jilin University, 126 Xiantai St., Changchun, 130033 Jilin China; 2 Jilin Provincial Cardiovascular Research Institute, 126 Xiantai St., Changchun, 130033 Jilin China; 3 Department of Pediatric Neurology, The First Hospital of Jilin University, 71 Xinmin St., Changchun, 130021 Jilin China

**Keywords:** Human bone marrow mesenchymal stem cells, Exosomes, Wound healing, TGF-β/Smad signaling

## Abstract

**Background:**

Cutaneous wound healing represents a morphogenetic response to injury and is designed to restore anatomic and physiological function. Human bone marrow mesenchymal stem cell-derived exosomes (hBM-MSC-Ex) are a promising source for cell-free therapy and skin regeneration.

**Methods:**

In this study, we investigated the cell regeneration effects and its underlying mechanism of hBM-MSC-Ex on cutaneous wound healing in rats. In vitro studies, we evaluated the role of hBM-MSC-Ex in the two types of skin cells: human keratinocytes (HaCaT) and human dermal fibroblasts (HDFs) for the proliferation. For in vivo studies, we used a full-thickness skin wound model to evaluate the effects of hBM-MSC-Ex on cutaneous wound healing in vivo.

**Results:**

The results demonstrated that hBM-MSC-Ex promote both two types of skin cells’ growth effectively and accelerate the cutaneous wound healing. Interestingly, we found that hBM-MSC-Ex significantly downregulated TGF-β1, Smad2, Smad3, and Smad4 expression, while upregulated TGF-β3 and Smad7 expression in the TGF-β/Smad signaling pathway.

**Conclusions:**

Our findings indicated that hBM-MSC-Ex effectively promote the cutaneous wound healing through inhibiting the TGF-β/Smad signal pathway. The current results provided a new sight for the therapeutic strategy for the treatment of cutaneous wounds.

## Introduction

Cutaneous wound healing is characterized by repairing damaged tissue, and the tissue regeneration was orchestrated by multiple cells to re-establish a protective barrier [1]. The primary goals of skin wound treatment consider rapid wound closure and scar-less healing process. Previous studies have shown that mesenchymal stem cells (MSCs) are capable of self-renewal and multipotential differentiation capabilities, which shows promising therapeutic potential for tissue regeneration and skin function recovery [2, 3]. However, MSC transplantation may induce immunoreactivity to the host [4]. To avoid host-immunoreactivity, MSC-derived exosomes shows an alternative therapeutic attention for regenerative wound repair [5–7]. The current study has demonstrated that MSC-derived exosomes accelerated the wound healing through increased re-epithelialization, angiogenesis, and tissue granulation [6]. In our previous publication, we have demonstrated that hBM-MSC-Ex treatment significantly reduced liver fibrosis in rats [8]. In this current study, we used the promising exosomes type, i.e., hBM-MSC-Ex, to investigate the wound healing process and to investigate the associated signal pathway mechanism.

TGF-β/Smad signal pathway is an evolutionarily conserved pathway with numerous functions ascribed [9]. Transforming growth factor-beta (TGF-β) is considered as one of the essential growth factors in the natural wound healing process by TGF-β/Smad pathway [10]. Smad is well known as the major inducer of fibroblast differentiation, which is an essential factor for wound healing [11]. During this process, TGF-β activates downstream mediators such as Smad2 and Smad3, which result in the linage differentiation of fibroblasts into alpha-smooth muscle expressing (α-SMA) myofibroblasts [12]. The phosphorylated Smad2/Smad3 activates Smad7, which promoter to upregulate Smad7 expression in the differentiation process, and thereby, Smad7 inhibits TGF-β1 expression for negative feedback regulation [13]. Recent studies have demonstrated that the TGF-β/Smad signal pathway plays an essential vital role in cutaneous wound healing via regulating the proliferation and migration of keratinocytes, dermal fibroblasts, and other skin cells in the affected areas to participate in the wound healing process [14, 15]. Furthermore, TGF-β/Smad signaling can regulate tissue fibrosis and scar formation [16]. In this current study, we hypothesized that hBM-MSC-Ex could promote the cutaneous wound healing process via regulating the TGF-β/Smad signaling pathway.

## Materials and methods

### Cell culture and exosome purification

HaCaT and HDFs were purchased from the Chinese Academy of Medical Sciences, China. Human bone marrow mesenchymal stem cells (hBM-MSCs) were generously provided by Dr. Yi Wang (Jilin University, Changchun, China), P3-5 lines of hBM-MSCs were used in the experiments. Cells cultured in DMEM (Gibco, Grand Island, USA) supplemented with 10% FBS (Gibco, Grand island, USA) humidified 5% CO_2_ atmosphere at 37 °C. The purification of hBM-MSC-Ex involves several centrifugation steps, as described previously [17]. Briefly, hBM-MSCs were cultured in serum-free medium (SFM, Gibco, Grand Island, USA) for 2 days. Conditioned medium was first filtered using a 0.1-μm filtering unit. The supernatant was concentrated with a 100-kDa molecular weight cutoff (MWCO) hollow fiber membrane (Millipore, Billerica, MA, USA), at 1000*g* for 30 min. Then, the concentrated supernatant was loaded onto a 30% sucrose/D_2_O cushion (5 ml, density 1.210 g/cm^3^), and ultra-centrifuged at 100,000*g* for 3 h. After exosome-enriched fraction was collected, it was washed three times with fresh PBS, centrifuged at 1500*g* (30 min each wash) with 100-KDa MWCO. Finally, purified exosomes were passed through a 0.22-μm filter and stored at − 80 °C until further use. The protein concentration of exosomes was measured by bicinchoninic acid (BCA) protein assay kit (Beyotime, Shanghai, China).

### Cell proliferation assay

HaCaT and HDFs cells were cultured until 70~80% confluence, and trypsinized cells were plated in 96-well plates at a density of 4000 cells per well. Briefly, hBM-MSC-Ex purification and characterization were as per previously published methods [8]. The cells were treated with either hBM-MSC-Ex (25 μg/mL) or PBS (control) (Invitrogen, Shanghai, China), then followed by incubated at 37 °C with 5% CO_2_ for 5 days. The cell viability was determined by 10% CCK-8 solution (Sigma, San Francisco, USA), the cultures were incubated for 30 min at 37 °C in 5% CO_2_, and corresponding OD value was measured at the 490-nm wavelength.

### Immunofluorescence staining (IF)

HaCaT and HDFs cultured by hBM-MSC-Ex (25 μg/mL) or PBS were incubated in 24-well plate coated coverslip for 24 h. When cells reached 60~70% confluence, plate were washed with PBS and incubated with 4% paraformaldehyde for 10 min (RT). The processed cells blocked with 1% bovine serum albumin (BSA; Biosharp, Hefei, China) for 30 min. The cells were incubated with primary antibodies against Rb anti-PCNA (1:100 dilution, BD Biosciences, Franklin Lakes, NJ, USA), and isotype-matched rabbit IgG/IgM (1:100 dilution, Abcam, Cambridge, UK) served as the negative controls. Anti-rb-FITC-488 secondary antibody (1:500 dilution, Abcam, Cambridge, UK) for 2 h, and the nuclei were labeled with DAPI (Thermo Scientific, Waltham, USA) for 5 min. Images were acquired by fluorescence microscopy (EVOS, Thermo Scientific, Waltham, USA), and the PCNA positive cells were counted in ten random optical fields by using ImageJ software.

### Animals and treatments

The 8-week-old female Sprague-Dawley (200 g) rats were purchased from Jilin Biotechnology Co., Ltd. (Changchun, China). All animal experiments were performed in accordance with the guidelines of the Animal Experiment Ethics Committee of Jilin University. The animal model was generated according to previously published methods [18]. Briefly, rats were anesthetized and the dorsal hair was shaved; following this, full-thickness skin excisional wound was made about the size of 10 mm in diameter circular holes in rat. The rats were randomly divided into three groups (*n* 8/group): the PBS group, hBM-MSC group (intravenous injection with 1 × 10^6^ cells/ rat), and hBM-MSC-Ex group (250 μg, multi-directional subcutaneous injection). The recovery of skin damage was recorded photographically every 4 days for 16 days. The wound area was measured using lasso tool (Adobe Photoshop CS6). The wounded area was traced, and we circled the edge of wound on photograph, then calculate the circled area based on the pixels of that area. At the end of the study, the rats were euthanized on the 16th day to collect the healed and unhealed tissue area in the different treatment groups.

### Histological examination

Skin tissue was collected from the mechanical injury region on 16 days, and samples were fixed in 10% formalin in PBS and embedded in paraffin. The skin tissue was sectioned at 4-μm thickness to perform hematoxylin and eosin (H&E) staining. The staining was performed following the manufacturer’s protocols (Sigma, San Francisco, USA). The sections were processed for immunohistochemistry (IHC) using the Kit (Maixin KIT-9710, Fuzhou, China) following the manufacturer’s instructions. Briefly, the sections were deparaffinized, and antigen retrieval was performed by immersion slides in 0.01 M sodium citrate buffer solution for 15 min. Endogenous peroxidase was quenched by processing the sections in 3% H_2_O_2_ for 15 min, followed by blocked sections with 10% normal goat serum for 1 h at 37 °C. The sections were incubated with primary antibody anti-α-SMA or anti-VEGF with 1:500 dilution (Abcam, Cambridge, UK) for overnight at 4 °C. Next day, these sections were incubated with biotinylated goat-anti-rabbit IgG antibody for 2 h and incubated with avidin peroxidase reagent sequentially. Then, the sections were incubated with diaminobenzidine solution as the chromogenic agent at 37 °C for 5 min. Finally, we used hematoxylin staining for counterstaining the sections. These sections were photographed using a bright-field microscope (EVOS, Thermo Scientific, Waltham, USA). Cutaneous appendage separated by tissue on one microscope sphere was counted as one unit. The α-SMA- and VEGF-positive area calculation is based on the ratio of positive area/total area of the observed field. We used 6 random fields per section and 6 sections in total (*n* = 8 rats) for the quantification of IHC images.

### Western blot

Proteins were extracted from the skin healed tissue in the in SDS sample lysis buffer. The samples were heated to 95 °C for 10 min, and 40-μg protein samples were separated on SDS-polyacrylamide gels (5% stacking gel and 12% separation gel). Resolved proteins were then transferred onto nitrocellulose membranes, blocked in 5% nonfat powdered milk for 1 h, and probed with respective antibody against TGF-β1, TGF-β3, Smad2, Smad3, Smad4, Smad7, and glyceraldehyde-3-phosphate dehydrogenase (GAPDH) served as loading control (1:1000 dilution, Abcam, Cambridge, UK) overnight at 4 °C. The blots were blocked in secondary HRP-conjugated goat anti-rabbit IgG antibodies (1:1000 dilution, Abcam, Cambridge, UK) and visualized by chemiluminescent detection substrates (Immobilon western chemiluminescent HRP substrate, Millipore). The densitometric quantification were performed on the protein bands by using AlphaEaseFC software (Alpha Innotech).

### Real-time PCR assay

Total RNA from skin healed tissue was extracted with Trizol (Invitrogen, Shanghai, China) according to the manufacturer’s protocol. Briefly, the first-strand cDNA was synthesized with 1 μg of total RNA using SuperScript II (Invitrogen). SYBR Green I dye was used for reverse transcription in an ABI 7500 fluorescence quantitative PCR instrument, and the mRNA levels of TGF-β1, TGF-β3, Smad2, Smad3, Smad4, Smad7, and GAPDH were measured using the respective primers listed in Table (Supplementary Table S)1. The thermocycler conditions were as follow: initial step at 95 °C for 2 min, followed by 40 cycles at 95 °C for 15 s, and 60 °C for 1 min. Expression levels were recorded as cycle threshold (Ct). Data were acquired using the 7500 Software (Applied Biosystems Life Technologies, Foster City, CA, USA). All reactions were performed in triplicate, and the data were analyzed using the 2^−ΔΔCt^ method.

### Statistical analysis

Statistical analysis was performed using Prism 6 (Graph Pad software) and Image J. One-way ANOVA with post hoc Dunnett’s multiple comparison test was used to test for statistically significant differences between the groups. All quantitative data were given as the mean ± SD, and data were acquired by at least three independent experiments, and *p* < 0.05 was considered to be statistically significant.

## Results

### In vitro studies: hBM-MSC-Ex improves skin cell proliferation

**Fig. 1:**
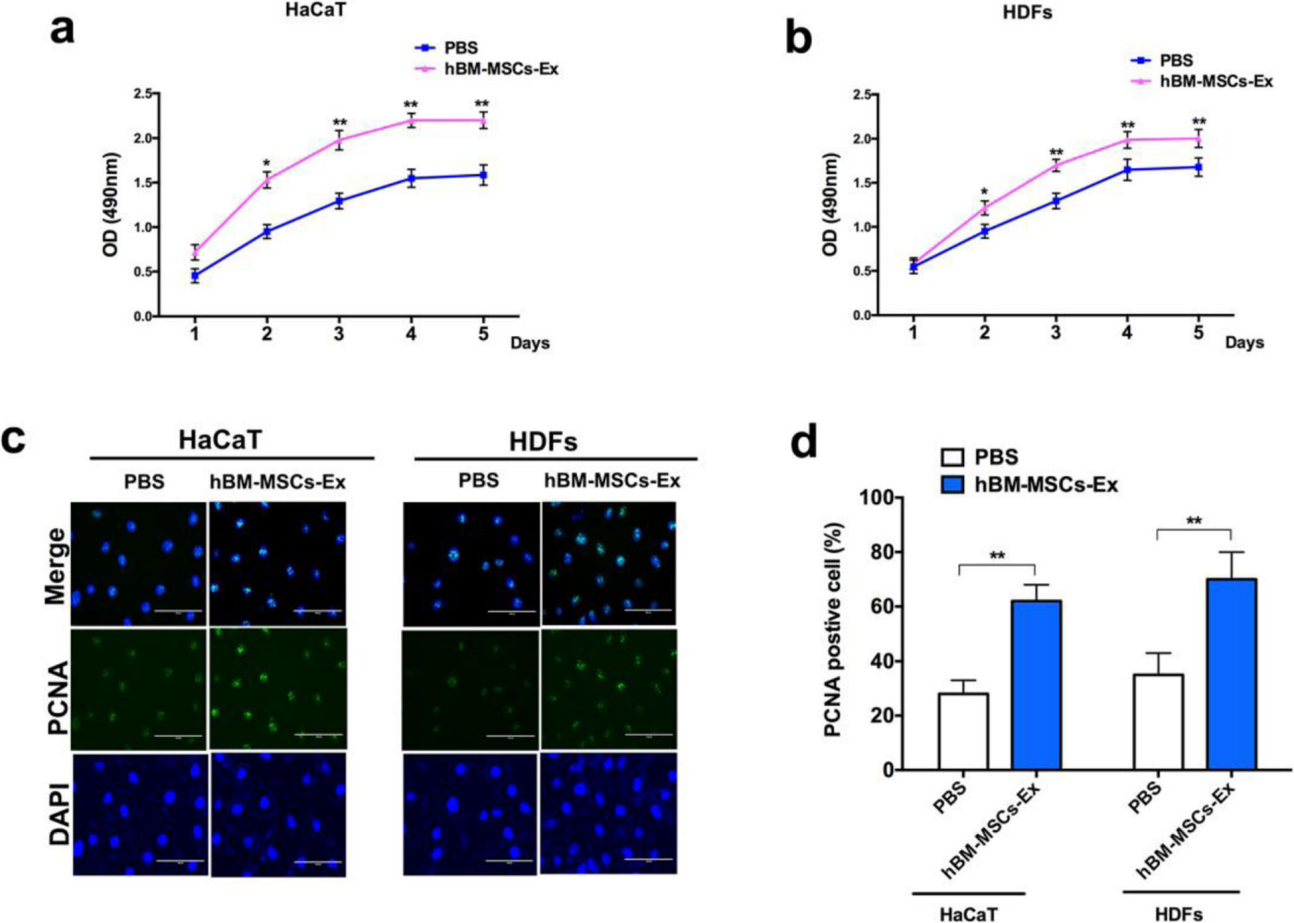
hBM-MSC-Ex promotes proliferation in HaCaT and HDF skin cells. The cell proliferation curve were shown from respective HaCaT and HDF skin cells after treated with hBM-MSC-Ex (**a**, **b**). Immunofluorescent staining was performed in HaCaT and HDFs for PCNA-positive cells, and representative images were shown (**c**). The quantification of PCNA-positive cells (in percentages) in HaCaT and HDFs were determined (**d**). HaCaT, human keratinocytes; HDFs, human dermal fibroblasts; bar = 100 μm, *p < 0.05, **p < 0.01, *n* = 3; data are reported as mean ± SD

In vitro, we investigated whether hBM-MSC-Ex treatment can stimulate the cell proliferation by using HaCaT and HDFs skin cells. After the procedure, the proliferation assay was measured consecutive for 5 days. In HaCaT and HDFs cells, we observed significant increase in exponential cell proliferation and the growth rate constantly increased from day 2 after the hBM-MSC-Ex treatment, compared to the PBS group (Fig. 1a, b, *p* < 0.05 and *p* < 0.01). Based on the CCK-8 results, we performed immunofluorescent staining analysis on day 5 to confirm whether hBM-MSC-Ex treatment can induce the proliferative effect in these cell lines. As shown in the representative images in Fig. 1c and d, then we observed a significant increase in the number of PCNA-positive cells in the hBM-MSC-Ex treatment group compared to the PBS group (62.1% vs 28.2% in HaCaT and 71.3% vs 34.8% in HDFs in the two types of skin cells (*p* < 0.01)). In vitro experiment, the results demonstrated that treatment of hBM-MSC-Ex can promote cell proliferation and maintain cell growth effectively in the both skin cells (HaCaT and HDFs).

### In vivo studies: hBM-MSC-Ex improves cutaneous wound healing

**Fig. 2:**
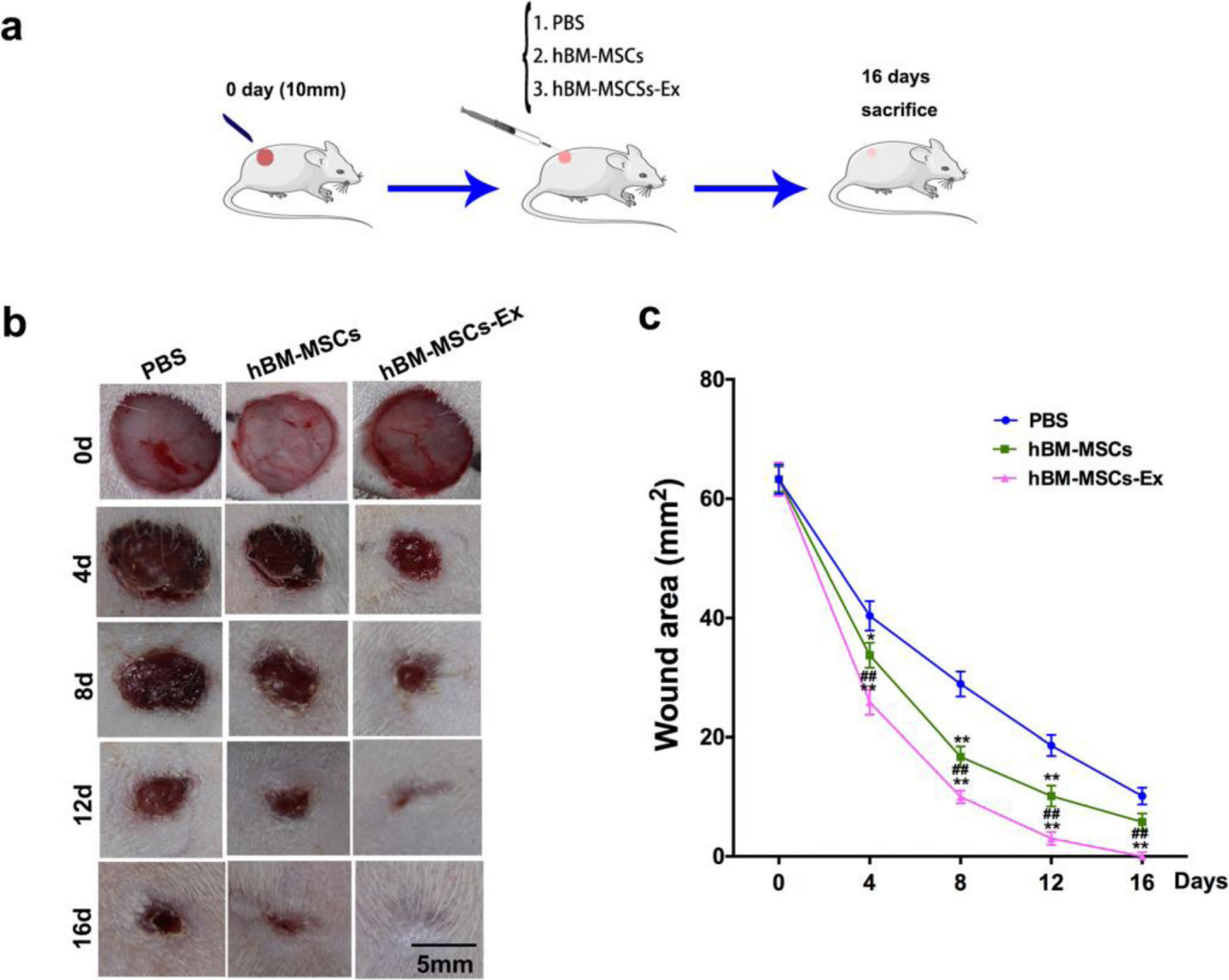
hBM-MSC-Ex treatments accelerate cutaneous wound healing process in vivo. The illustration of experimental design and plan of experiment performed in vivo (**a**). The representative photos shown in the dorsal full-thickness wound area of the rat (**b**). Quantitative analysis of wound area in the respective treatment groups (**c**). *n* = 8/group, scale bar = 5 mm, *p < 0.05, **p < 0.01 compared to the PBS group, ^##^*p* < 0.01 compared to the hBM-MSC group; data are reported as mean ± SD

To investigate the roles of hBM-MSC-Ex in wound healing, we established a full-thickness skin wounds injury model in rats, as illustrated in the experimental design in Fig. 2a. The area measurements in the wounded region show a significant improvement in the wound closure after the treatment of hBM-MSC-Ex and hBM-MSCs treatment group (Fig. 2b, c). We observed a significant reduction in the wound area in the hBM-MSCs treatment group starting from day 4 after the surgery and constantly improved in reduction until the day 16 compared with PBS. The wound in hBM-MSC-Ex group completely healed on the 16 ± 2.3 days after surgery (*p* < 0.05, *p* < 0.01). These results indicates that hBM-MSCs’ isolated exosomes can more effectively accelerated the cutaneous wound healing process as demonstrated in the animal model.

### hBM-MSC-Ex restore the normal skin morphology

**Fig. 3:**
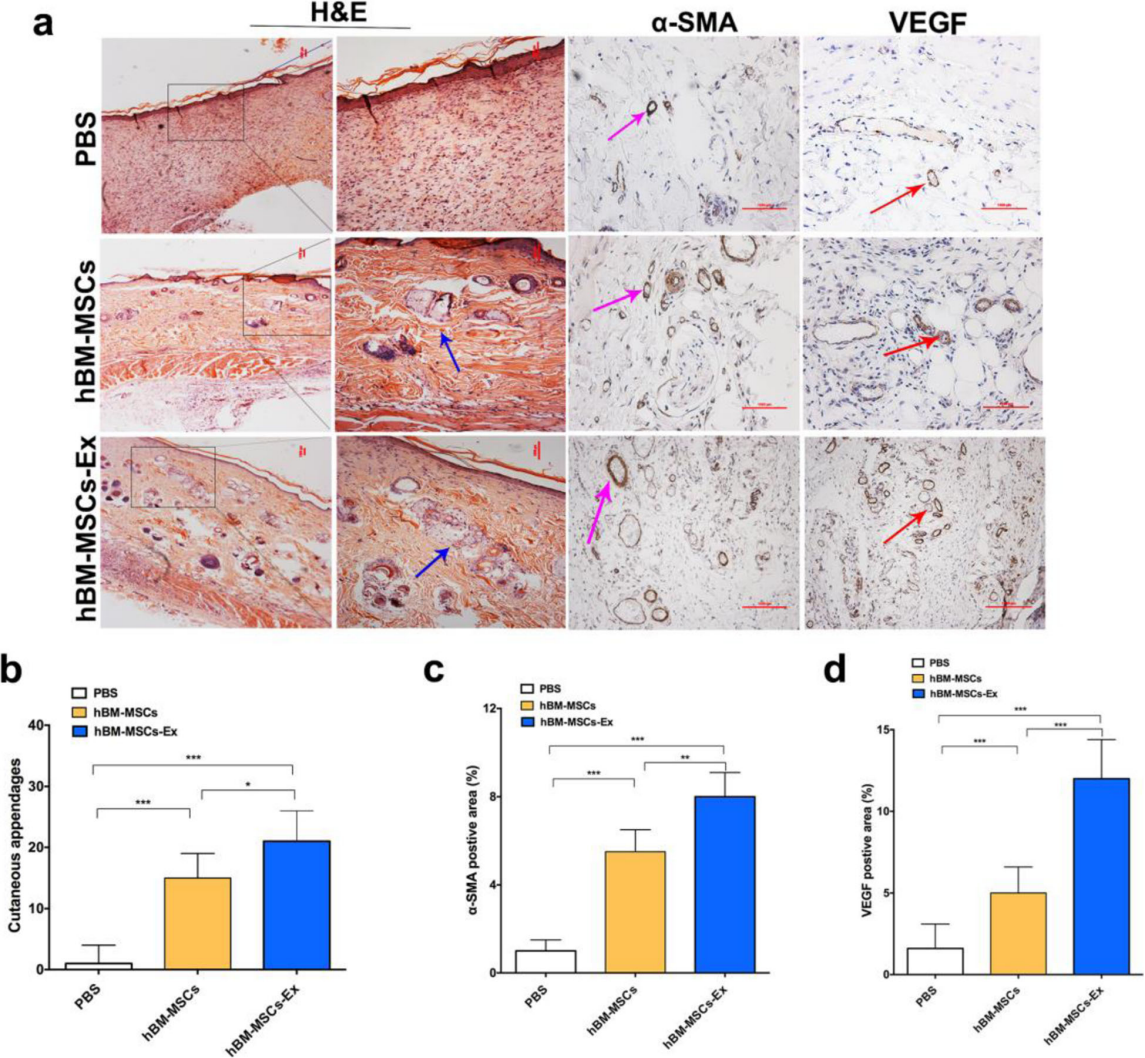
hBM-MSC-Ex enhanced the cutaneous wound healing quality. The representative images of H&E staining in the different treatment groups (**a**). The image quantification of number of cutaneous appendages including hair follicles and sebaceous glands/field (40×) in the healing tissue (**b**). The blue arrow indicates the presence of sebaceous gland. Immunostaining for α-SMA and VEGF were shown respective images at 16 days after treatment (**a**). The percentage quantification of α-SMA and VEGF positive area (**c**, **d**). The purple arrow indicates the α-SMA-positive cells, and red arrow indicates VEGF positive cells. Scale bar = 1 mm, *p < 0.05, **p < 0.01, ***p < 0.001, *n* = 8/group, data are reported as mean ± SD

To understand the process of restoring the skin morphology regenerated during the healing process, we assessed the effects of the hBM-MSC-Ex on the wound healing quality. To study the general morphology of skin regeneration, we performed H&E staining. The observation of H&E staining images shows an indication that regeneration of cutaneous appendages in the affected area, including restoring hair follicles and sebaceous glands in hBM-MSC-Ex group (21.3 ± 5.4 /filed), was compared to other treatments such as the PBS group (1.2 ± 2.8/filed, *p* < 0.001) the and hBM-MSC group (15.4 ± 4.1 /filed, *p* < 0.001) (Fig. 3a, b). α-SMA and VEGF are both important indicators of angiogenesis. We performed IHC in the wound area samples from the different treatment groups. The α-SMA-positive area quantification results showed that the percentage of α-SMA positive area were significantly increased in the hBM-MSC-Ex group (8.1% ± 1.2), when compared with the PBS group (1.3% ± 0.5/HPF, *p* < 0.001) as well as the hBM-MSC group (5.6% ± 0.9, *p* < 0.01) (Fig. 3a, c). Consistent with the above results, the percentage of VEGF-positive area was significantly increased in the hBM-MSC-Ex group (12.3% ± 2.4), compared to the PBS group (1.6% ± 1.5/HPF, *p* < 0.001) and the hBM-MSC group (5.1% ± 1.7, *p* < 0.001) (Fig. 3a, d). The above results indicated that hBM-MSC-Ex not only enhanced the wound process but also restored skin function and angiogenesis in the affected areas.

### hBM-MSC-Ex regulate the TGF-β/Smad signal pathway

**Fig. 4:**
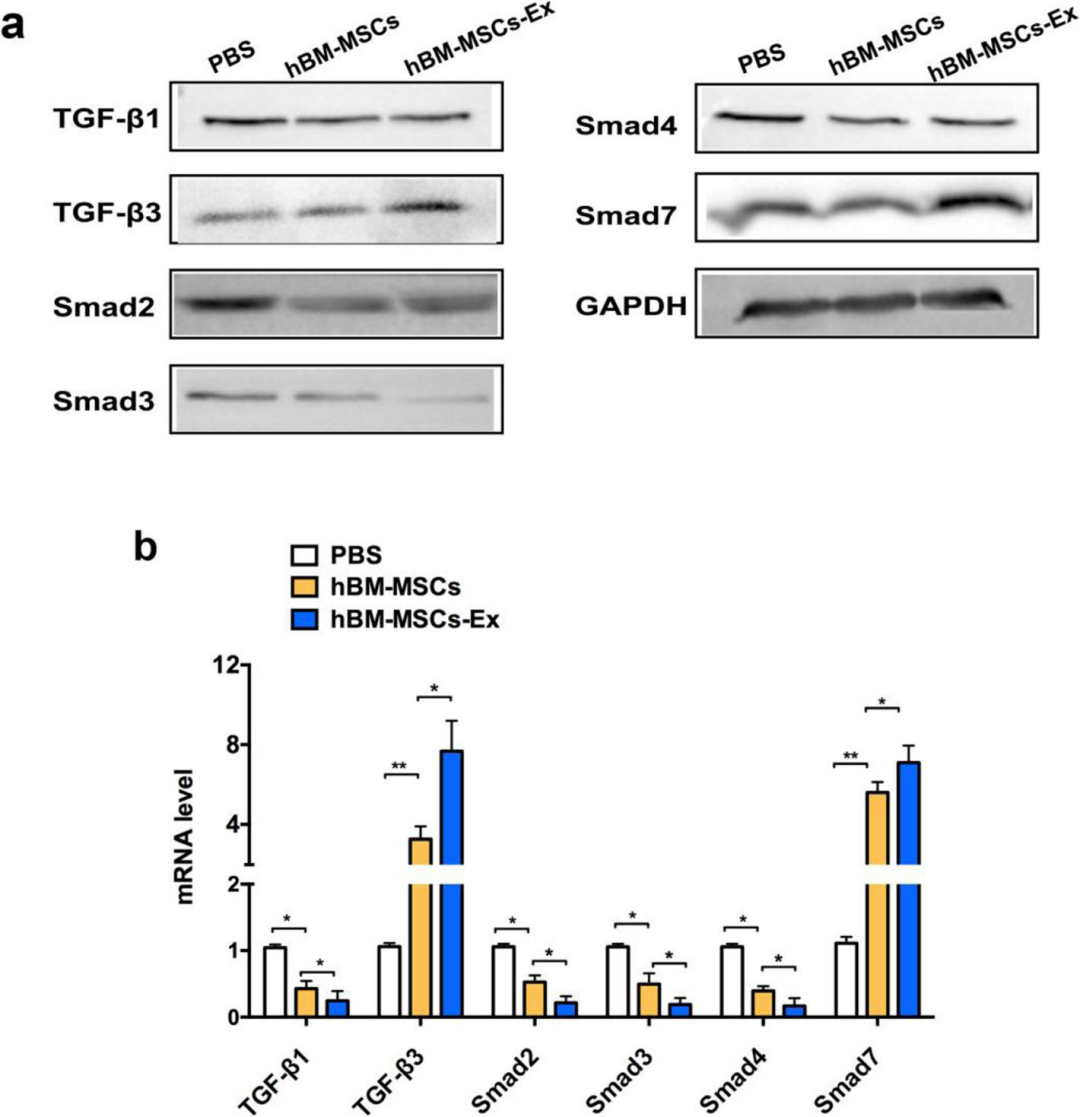
hBM-MSC-Ex regulate the TGF-β/Smad signal pathway. Representative Western blot of key TGF-β/Smad signaling-related protein levels in skin tissue treated with hBM-MSC-Ex (**a**). The quantification of relative mRNA expression levels of major TGF-β/Smad signaling-related gene in skin healed tissue treated with hBM-MSC-Ex (**b**). *p < 0.05, **p < 0.01, *n* = 3, data are reported as mean ± SD

Finally, we investigated the underlying mechanism of hBM-MSC-Ex-induced skin healing process in the affected tissue. The expression level of TGF-β1, TGF-β3, Smad2, Smad3, Smad4, and Smad7 (components of the TGF-β/Smad signaling pathway) were analyzed by Western blot and RT-qPCR. Both Western blot and RT-qPCR results showed the significantly decreased (respective protein and RNA levels) expression of TGF-β1, Smad2, Smad3, and Smad4 in hBM-MSC-Ex treatment group, compared with the other two control groups (*p* < 0.05, *p* < 0.01). The alteration of signaling molecule during the healing the process illustrated that hBM-MSC-Ex treatment plays a major mechanism in inhibiting TGF-β/Smad signaling pathway (Fig. 4a, b).

**Fig. 5:**
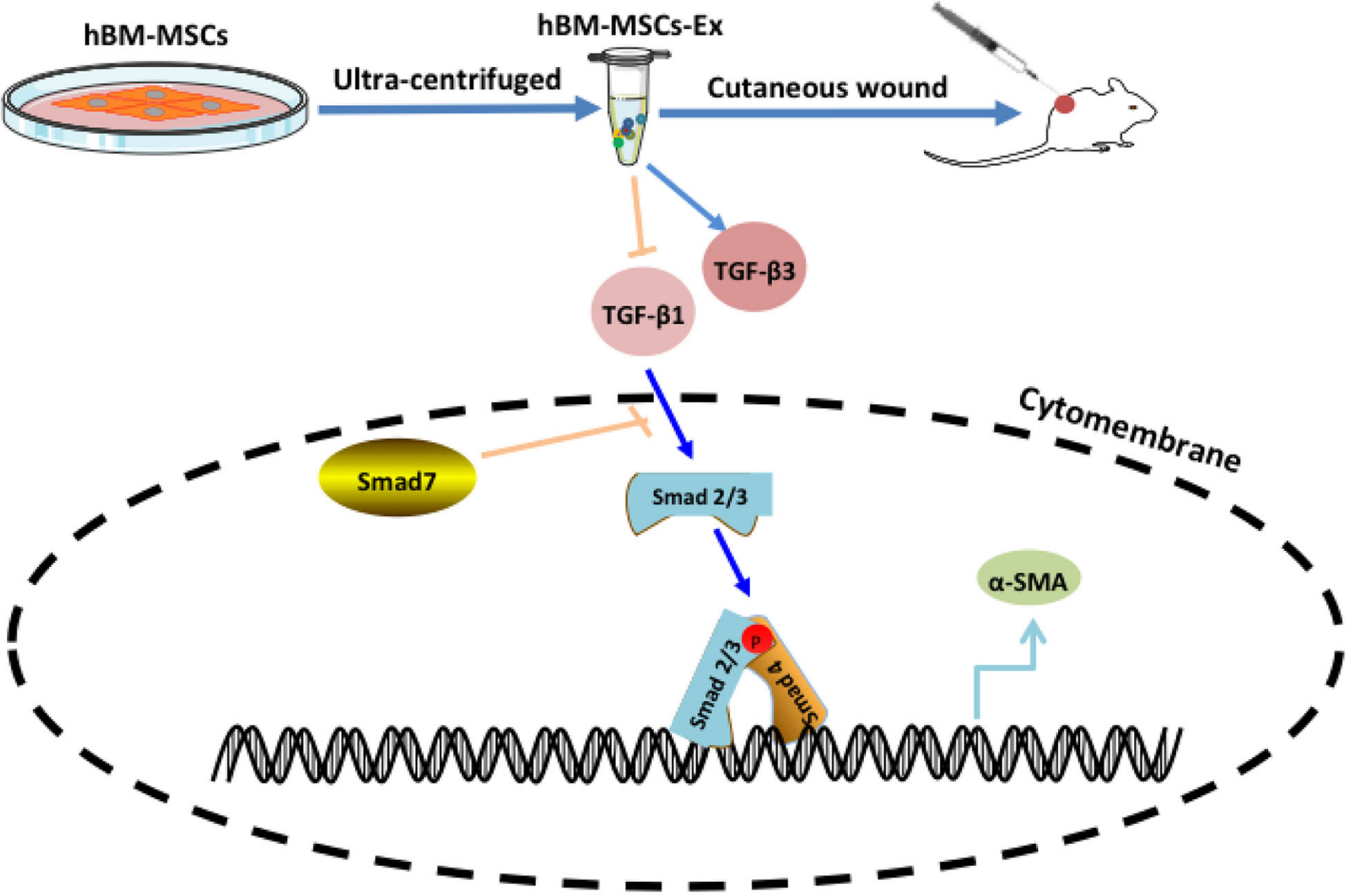
Illustration of hBM-MSC-Ex stimulates cutaneous wound healing by regulating the TGF-β/Smad signal pathway. hBM-MSC-Ex inhibited TGF-β1 and activated TGF-β3 expression; TGF-β isoforms and activins stimulate intracellular signaling via Smad-2/3 transcription factors; phosphorylated Smad-2 and Smad-3 bind to Smad-4 leading to the transcription and expression of α-SMA; inhibitory Smad7 are activated by the binding of the TGF-β super family to the cell surface receptors

In the current study, we aimed to study how the TGF-β/Smad signal modulated during wound healing. In our result, we detected that Smad7 levels were significantly increased in the hBM-MSC-Ex-treated group compared to other treatment groups (Fig. 4a, b; *p* < 0.05, *p* < 0.01). These results suggested that altering the levels of Smad7 could be beneficial in the wound regeneration process. Inhibitory Smad7 is activated by the binding of the TGF-β super family to the cell surface receptors. TGF-β1 is fibrotic isoform, while TGF-β3 is the anti-fibrotic isoform. The hBM-MSC-Ex treatment group significantly upregulated the expression of TGF-β3, compared with the other two control groups (Fig. 4a, b; *p* < 0.05, *p* < 0.01). These results suggest that hBM-MSC-Ex effectively promote the cutaneous wound healing through inhibiting the TGF-β/Smad signal pathway (Fig. 5).

## Discussion

In this study, our results indicate that hBM-MSC-Ex stimulates cutaneous wound healing both in vitro and in vivo*.* In vitro, hBM-MSC-Ex promotes both two types of skin cell (HaCaT and HDFs) proliferation effectively. In vivo, hBM-MSC-Ex accelerates cutaneous wound healing, via inhibiting the TGF-β/Smad signal pathway.

Recently, studies have shown that the prospective application of MSC-derived exosomes promoted cutaneous wound healing [5, 7, 19]. The possible roles of MSC-derived exosomes in wound healing are promotion of cell proliferation, migration, differentiation, angiogenesis, and matrix reconstruction [6]. HaCaT and HDFs are the two major skin cells types which participate in cutaneous wound healing [20]. In our study, we found that hBM-MSC-Ex promote HaCaT and HDFs growth effectively (Fig. 1). It indicated that hBM-MSC-Ex promote HaCaT and HDF cell proliferation to participate in the process of wound healing. In addition, fibroblasts are critical players for exosomes in wound healing and are the main cell types that synthesize, secrete, and deposit ECM collagen and elastic fibers [21]. Studies have shown that MSCs exert their therapeutic effect via secretion of soluble factors, including the exosomes [5, 6]. There is substantial evidence that exosomes have the ability to promote skin regeneration when applied topically or injected systemically [22, 23]. In this study, we have administrated subcutaneous injection of exosomes at multi-directional to delay its clearance in the body. In addition, hBM-MSCs appear to be limited because of poor cell retention at the wound site [24], To overcomes this issue, we adopted intravenous injection routes to maximize the effect of treating wounds in our treatment groups.

MSC-derived exosomes accelerate wound healing process through promoting angiogenesis and restoration of skin function [25]. Previous studies have proved that stem cell-conditioned medium may contain exosomes that contain pro-angiogenesis factors to promote wound healing in skin injury [26, 27]. Our results demonstrate that hBM-MSC-Ex significantly accelerate wound healing (Fig. 2b, c) and modulate α-SMA expression which is an important indicator of angiogenesis (Fig. 3a, c). MSC-derived exosome contains various growth factors which play an important role in cutaneous regeneration and repair [6, 19]. Interestingly, we also found there are many cutaneous appendages regeneration, such as hair follicles and sebaceous glands (Fig. 3a, b). It provides favorable conditions for hBM-MSC-Ex to restore skin function during wound healing.

Recent studies indicated that different types of MSC therapy or combined therapies with some unique biotechnology factors can stimulate cutaneous wound healing [28–31]. Current studies have report that a new combination therapeutical approach shows effective combinational treatment composed of adipose-derived mesenchymal stem cells (AD-MSCs) platelet-rich plasma (PRP) and hyaluronic acid (HA) dressing which shown to stimulate the wounds healing and regeneration process [30–32]. The complete closure of wound is inducing a new neodermis and stimulating regeneration and a protected environment in a humid environment [33–35]. Many supporting evidence indicates that adipose-derived stem cells (ASCs) and adipocyte-secreted exosomal microRNA promote wound repair [36]. Another study has demonstrated hBMSCs on gelatin scaffold with poly *N*-isopropylacrylamide (pNIPAAm) as transplanted grafts for improving skin regeneration [37]. More recent clinical trials have shown to improve the hair density by administration human follicle stem cells (HFSCs) [38–40]. In addition, autologous fat grafting is a better approach to consider for the correction of wound scars in the affected regions [41, 42]. However, in our current study, we administrated directly hBM-MSC-Ex on mechanical damaged skin area, which is more clinically transferable, more safe, and effective cell-free reagents compared with their parents’ cell skin regeneration therapies. Moreover, we have demonstrated that hBM-MSC-Ex treatment is more effective than of hBM-MSCs in wound healing, such as some indicators of wound area and cutaneous appendages.

TGF-β1/Smad pathway is an important pathogenic mechanism in wound healing [9]. TGF-β1 is considered to be a key mediator in tissues scarring and mostly by activating its downstream against decapentaplegic (Smad) signaling [13]. It has proven that TGF-β1 exerts its biological effects by activating downstream mediators, including Smad2 and Smad3 [15]. The phosphorylated cytoplasmic mediators, Smad2 and/or Smad3, and a heterotrimeric complex are formed with Smad4 that translocate into the nucleus, bind a consensus sequence, and regulate gene transcription [14], while these activities are negatively regulated by Smad7 expression [16]. In our study, we found hBM-MSC-Ex significantly downregulate TGF-β1, Smad2, Smad3, and Smad4 expression and upregulate of Smad7 expression (Fig. 4). It demonstrated that hBM-MSC-Ex might accelerate cutaneous wound healing through inhibiting the TGF-β/Smad signal pathway (Fig. 5). TGF-β1 are associated with fibrosis, while TGF-β3 has been associated with anti-fibrotic or scar-less wound healing activity, and they have been observed to play an essential role in regulating epidermal and dermal cell movement during wound repair [10]. In our study, we found hBM-MSC-Ex decreased of TGF-β1 expression and increased TGF-β3 expression (Fig. 4). This may be one of the crucial reasons to promote the skin scar-less wound healing.

In conclusion, we successfully investigated the role of hBM-MSC-Ex on cutaneous wound healing. Our results demonstrated that hBM-MSC-Ex could exert promoting effect of cutaneous wound healing via inhibiting the TGF-β/Smad signal pathway. The current approach provides a better knowledge in the wound healing process and new therapeutic strategy for the treatment of cutaneous wounds.

## Supplementary information

**Additional file 1: Table S1.** Primers used for qRT-PCR.

## Data Availability

The datasets used and/or analyzed during the present study are available from the corresponding author on reasonable request.

## Abbreviations

hBM-MSCs: Human bone marrow-derived mesenchymal stem cells
hBM-MSC-Ex: Human bone marrow mesenchymal stem cell-derived exosomes
ALP: Alkaline phosphatase
ALT: Alanine aminotransferase
AST: Aspartate aminotransferase
HSCs: Hepatic stellate cells
Hyp: Hydroxyproline
MDA: Malonaldehyde
qRT-PCR: Quantitative real-time PCR
HaCaT: Human keratinocytes
HDFs: Human dermal fibroblasts
TBIL: Total bilirubin
TP: Total protein
α-SMA: Alpha-smooth muscle actin
VEGF: Vascular endothelial growth factor
γ- GT: Gamma glutamyl transpeptidase
AD-MSCs: Adipose derived mesenchymal stem cells
PRP: Combined treatment composed of platelet-rich plasma
HA: Hyaluronic acid
pNIPAAm: *N*-Isopropylacrylamide
HFSCs: Human follicle stem cells
