# Supplementary material for: Human bone marrow mesenchymal stem cell-derived exosomes stimulate cutaneous wound healing mediates through TGF-β/Smad signaling pathway
Source: Stem Cell Res Ther. 2020 May 24;11:198. doi: 10.1186/s13287-020-01723-6 (PMC7245763; doi:10.1186/s13287-020-01723-6)
Supplement: Supplementary file 1 — Additional file 1: Table S1. Primers used for qRT-PCR. [file 13287_2020_1723_MOESM1_ESM.docx]

**Table S1. Primers used for** **qRT-PCR**

| Gene | Primers | Sequences | Product size (bp) |
| --- | --- | --- | --- |
| GAPDH | Forward | CCGAGACATCAAGGAGAAG | 151 |
|  | Reverse | GTAGTTTCGTGAATGCCGCA |  |
| TGFβ1 | Forward | GGACGCCGTAAGTGCTTTGA | 143 |
|  | Reverse | GGACGAACGACTAGGTGTAG |  |
| TGFβ3 | Forward | CATCTACAAGAAGTTGGAGC | 165 |
|  | Reverse | CAAACACGCACCTCAAAGC |  |
| Smad2 | Forward | CCCTCCCAGAAGACCTACC | 138 |
|  | Reverse | GGCACAAACACGCACCTCA |  |
| Smad3 | Forward | GCTAACCCTTCGCTCCGT | 125 |
|  | Reverse | GCTTCCTCCTCTTTATCAG |  |
| Smad4 | Forward | TTAGTGGTGATGCCTTTG | 141 |
|  | Reverse | TCCCATGTACTGTCCTCC |  |
| Smad7 | Forward | CTCAGAACCTGCTTATCAA | 132 |
|  | Reverse | CTGGCTTGGTCACATCTTG |  |
|  |  |  |  |
